# Analysis of time-dependent changes in the FIB4 index in patients with obesity receiving weight reduction therapy

**DOI:** 10.1038/s41598-022-19420-0

**Published:** 2022-09-08

**Authors:** Shiori Kawai, Hajime Yamakage, Kazuhiko Kotani, Mitsuhiko Noda, Noriko Satoh-Asahara, Koshi Hashimoto

**Affiliations:** 1 Department of Diabetes, Endocrinology and Hematology, Dokkyo Medical University Saitama Medical Center, 2-1-50 Minami-Koshigaya, Koshigaya, Saitama 343-8555 Japan; 2 Department of Diabetes Mellitus, Saitama Cooperative Hospital, 1317 Kizoro, Kawaguchi, Saitama 333-0831 Japan; 3 Division of Diabetic Research, Clinical Research Institute, National Hospital Organization Kyoto Medical Center, 1-1 Fukakusa Mukaihata-cho, Fushimi-ku, Kyoto, 612-8555 Japan; 4 Division of Community and Family Medicine, Jichi Medical University, 3311-1 Yakushiji, Shimotsuke, Tochigi 329-0498 Japan; 5 Department of Diabetes, Metabolism and Endocrinology, Ichikawa Hospital, International University of Health and Welfare, 6-1-14 Kounodai, Ichikawa, Chiba 272-0827 Japan; 6 Department of Metabolic Syndrome and Nutritional Science, Research Institute of Environmental Medicine, Nagoya University, Nagoya, Aichi 464-8601 Japan

**Keywords:** Biomarkers, Gastroenterology, Health care, Medical research

## Abstract

Weight reduction therapy represents a fundamental strategy to prevent nonalcoholic fatty liver disease (NAFLD) in patients with obesity, which may result in liver fibrosis. Histological findings previously demonstrated that weight reduction therapy attenuated NAFLD. The FIB4 index is widely used to assess the status of NAFLD. The present study investigated whether the FIB4 index improved during weight reduction therapy. We used cohort data of the Japan Obesity and Metabolic syndrome Study and examined the correlation between body weight (BW) loss (BW loss) and changes in the FIB4 index (ΔFIB4 index) in patients who successfully reduced their BW by more than 5% from baseline BW after 3, 6, and 12 months (M) of weight reduction therapy. A negative correlation (r = −0.342, p = 0.029) was observed between BW loss and FIB4 index after 3 M, but not after 6 M, whereas a positive correlation (r = 0.298, p = 0.03) was noted after 12 M. These results revealed changes in the correlation between ΔBW loss and ΔFIB4 index during the therapy, mainly due to time-dependent changes in components of the FIB4 index formula. Thus, we concluded that the FIB4 index is useful and reliable to assess liver fibrosis until 3 M during weight reduction therapy. However, after 3 M, we should recognize that the FIB4 index may not reflect liver status. Therefore, it is important to consider this characteristic of the FIB4 index as a limitation when assessing liver fibrosis in obese patients receiving weight reduction therapy.

## Introduction

Nonalcoholic fatty liver disease (NAFLD), also known as metabolic-associated fatty liver disease (MAFLD), is a leading cause of chronic liver disease worldwide^1,2^. It encompasses a spectrum of liver diseases that range from hepatic steatosis to nonalcoholic steatohepatitis (NASH), which may progress to cirrhosis with advanced fibrosis^3–5^. Lifestyle modifications with the goal of weight loss represent a fundamental and important strategy to prevent NAFLD in patients with obesity^6,7^. Previous studies examined the impact of weight loss on histological changes in the liver and reported histological improvements in steatosis in 65% of obese patients with NAFLD who achieved body weight (BW) loss of more than 5% from the baseline^8^. Furthermore, significant histological improvements in fibrosis were observed in 45% of patients with obesity who achieved BW loss of more than 10% from the baseline^9^.

The ultimate goal of NAFLD treatment is to prevent hepatic fibrosis, which may progress to cirrhosis and, ultimately, hepatic carcinoma^10–12^. Although the gold standard examination for hepatic fibrosis is biopsy, non-invasive and simple tools are needed in daily practice^7^. The FIB4 index is a blood-based diagnostic test that is widely used to diagnose hepatic fibrosis^13^. It involves a highly sensitive, specific, and non-invasive scoring system to assess the status of NAFLD^13–16^.

The present study investigated whether the FIB4 index reflects the attenuation of hepatic fibrosis during weight reduction therapy and hypothesized that it may be improved by weight loss. Therefore, we used cohort data of the Japan Obesity and Metabolic syndrome Study (JOMS), a large-scale and multi-center study of Japanese patients with obesity receiving weight reduction therapy with both cross-sectional and prospective designs^17,18^. Since hepatic histological improvements were reported in patients with NAFLD who achieved BW loss of more than 5% from the baseline, as described above^8^, these patients were defined as those who succeeded in weight reduction therapy and we analyzed time-dependent changes in the FIB4 index (ΔFIB4 index) during weight reduction therapy. Patients were enrolled in JOMS and the present study was conducted as a series of the Kyoto Diabetes and Obesity Registry study (DOR-KyotoJ).

## Results

### Baseline characteristics of patients

Table 1 summarizes the baseline characteristics of the study cohort of 338 patients with obesity. The cut-off value for the FIB4 index to identify fibrosis was selected by age^19–21^. Therefore, we categorized patients into three age groups, as previously reported^19^ (Table 2, left column). No significant differences were observed in the numbers of males and females between the three groups. Since there are sex differences in metabolic parameters^22,23^, we also showed baseline data categorized by sex (Table 2, right column).

**Table 1:** Baseline characteristics of patients.

Variable	Total (n = 338)
Age (years)	52 (19–82)
Male/Female	155/183
BW (kg)	78.9 (53–168)
BMI (kg/m^2^)	30.1 (25–56)
WC (cm)	99 (71–150)
% fat (%)	36.0 (16.8–64.5)
SBP (mmHg)	140 (96–197)
DBP (mmHg)	85 ± 11.8
FPG (mg/dl)	101 (66–407)
HbA1c (%)	5.7 (4.5–11.6)
IRI (μU/ml)	14.8 (2.0–399)
HOMA-R	3.96 (0.55–170)
HOMA-β	140 (14.7–3048)
AST (IU/l)	24 (8–667)
ALT (IU/l)	29 (6–315)
Plt (10^9^/L)	259 (80–3010)
γ-GTP (IU/l)	40 (9–2527)
TG (mg/dl)	148 (47–1766)
HDL-C (mg/dl)	55 (18–103)
LDL-C (mg/dl)	129 ± 30.1
Leptin (ng/ml)	13.3 (0.96–136)
Adiponectin (μg/ml)	6.24 (1.29–36.1)
FIB4 index	0.86 (0.07–18.5)
**Concomitant medication at baseline, n (%)**	
Antidiabetic drugs, n (%)	77 (22)
Metformin	17
Thiazolidinediones	10
Sulfonylureas	52
Insulin	6
Antilipidemic drugs, n (%)	155 (46)
Statins	81
Fibrates	12
Antiobesity drugs, n (%)	21 (6)
**Alcohol consumption**	
Never, n (%)	147 (56)
Sometimes, n (%)	67 (25)
Every day, n (%)	49 (19)

Data are expressed as the mean ± SD, medians (min, max), or percentages. BW: body weight, BMI: body mass index, WC: waist circumference, SBP: systolic blood pressure, DBP: diastolic blood pressure, FPG: fasting plasma glucose, IRI: immunoreactive insulin, % fat: percentage total body fat, HOMA: homeostasis model assessment, AST: aspartate aminotransferase, ALT: alanine aminotransferase, Plt: platelet count, γ-GTP: γ-glutamyltransferase, TG: triglyceride, HDL-C: high-density lipoprotein-cholesterol, LDL-C: low-density lipoprotein-cholesterol.

**Table 2:** Baseline characteristics of patients categorized by age and sex.

Variable	Age ≤ 35 year (y) (n = 54)	36 y ≤ Age ≤ 64 y (n = 221)	65 y ≤ Age (n = 63)	p value	Male (n = 155)	Female (n = 183)	p value
Age (years)	28 ± 4.1	51 (36–64)	70 (65–82)	< 0.001*	51.0 (19–82)	52.0 (21–80)	–
Male/Female	26/28	98/123	31/32	–			
BW (kg)	98.2 ± 23.4	78.3 (53–132)	73.4 (57.8–128.1)	< 0.001*	85.9 (61–160)	73.6 (53–168)	< 0.001*
BMI (kg/m^2^)	36 ± 6.9	29.5 (25–52.9)	28.8 (25.0–45.4)	< 0.001*	30.0 (25–52.2)	30.2 (25–56.1)	–
WC (cm)	108 ± 16.8	98 (71–132)	100 (81–150)	< 0.001*	100 (71–150)	98.0 (71.5–141)	0.026*
% fat (%)	42.6 ± 10.3	35.4 (17.9–64.5)	33.5 ± 9.2	< 0.001*	30.4 (17.9–64.5)	39.4 (16.8–64.2)	< 0.001*
SBP (mmHg)	140 ± 19	140 (104–197)	141 ± 18	–	141 (108–197)	139 ± 18.6	–
DBP (mmHg)	86 ± 13	83 ± 11	82 ± 12	–	87 ± 12.1	83 ± 18.8	0.014*
FPG (mg/dl)	96.5 (74–269)	101 (66–407)	113 (68–131)	0.002*	103 (67–329)	99 (66–407)	–
HbA1c (%)	5.7 (5.4–6.1)	6.1(5.9–6.2)	6.1 (5.9–6.3)	< 0.001*	5.6 (4.5–11.6)	5.7 (4.6–10.3)	–
IRI (μU/ml)	45.7 (25.4–65.9)	22.5 (18.1–24.0)	22.4 (15.3–29.5)	< 0.001*	14.8 (3.3–398.8)	15.0 (2.0–255.9)	–
HOMA-R	14.5 (5.9–23.1)	6.3 (5.3–7.4)	7.6 (4.6–10.5)	0.017*	4.1 (0.65–170)	3.8 (0.55–111.8)	–
HOMA-β	391 (299–483)	221 (176–266)	171 (124–220)	< 0.001*	133.9 (25.3–1646)	143.0 (14.7–3048)	–
AST (IU/l)	27 (13–130)	24 (8–89)	25 (12–667)	–	26 (13–667)	21 (8–130)	< 0.001*
ALT (IU/l)	55 (14–171)	30 (6–149)	26 (10–315)	< 0.001*	34 (11–315)	26 (6–171)	< 0.001*
Plt (10^9^/L)	316 ± 62	258 (95–3010)	219 ± 61	< 0.001*	250 (130–402)	276 (80–3010)	< 0.001*
γ-GTP(IU/l)	56 (17–182)	40 (9–376)	31 (11–2527)	0.007*	55 (16–2527)	27 (9–251)	< 0.001*
TG (mg/dl)	204 (150–263)	181 (175–219)	158 (140–176)	–	223 (190–257)	164 (151–178)	0.002*
HDL-C (mg/dl)	51 ± 10.2	56 (55–59)	59 (55–63)	0.002*	51 (49–53)	61 (59–63)	< 0.001*
LDL-C (mg/dl)	130 ± 27.7	130 ± 29.2	124 ± 26.7	–	129 (124–134)	128 (124–133)	–
Leptin (ng/ml)	16.5 (3.7–130.9)	12.8 (1.0–135.7)	12.5 (1.6–88.7)	–	7.0 (1.0–88.4)	20.0 (2.2–135.7)	< 0.001*
Adiponectin (μg/ml)	4.76 (2.06–12.0)	6.30 (2.3–36.1)	7.2 (1.29–25.1)	< 0.001*	5.60 (1.30–18.9)	6.81 (2.30–36.1)	< 0.001*
FIB4 index	0.4 (0.37–0.46)	0.84 (0.07–3.34)	1.75 (0.78–18.5)	< 0.001*	0.95 (0.28–18.5)	0.83 (0.07–7.97)	–
**Concomitant medication at baseline, n (%)**							
Antidiabetic drugs, n (%)	6 (11)	56 (25)	15 (24)	–	36 (23)	40 (22)	–
Metformin	3	13	1	–	9	8	–
Thiazolidinediones	0	7	3	–	6	4	–
Sulfonylureas	5	36	11	–	25	27	–
Insulin	0	4	2	–	1	5	–
Antilipidemic drugs, n (%)	17 (31)	105 (48)	33 (52)	–	65 (42)	90 (49)	–
Statins	5	57	19	0.017*	29	52	0.037*
Fibrates	0	10	2	–	8	4	–
Antiobesity drugs, n (%)	5 (9)	14 (6)	2 (3)	–	7 (4)	14 (7)	–
Alcohol consumption				–			
Never, n (%)	28 (62)	95 (55)	24 (55)		54 (44)	93 (66)	< 0.001*
Sometimes, n (%)	14 (31)	44 (25)	9 (20)		32 (26)	35 (25)	
Every day, n (%)	3 (6)	35 (20)	11 (25)		37 (30)	12 (9)	

Data are expressed as the mean ± SD, medians (min, max), or percentages.

Asterisks indicate a significant difference (p < 0.05).

When patients were categorized by age, serum high-density lipoprotein-cholesterol (HDL-C) and adiponectin levels significantly increased, whereas BW, body mass index (BMI), percent body fat, immunoreactive insulin (IRI), the insulin resistance index (HOMA-R), insulin secretion index (HOMA-β), serum leptin and alanine aminotransferase (ALT) levels, and the platelet count significantly decreased in an age-dependent manner. No significant differences were observed in systolic (SBP) or diastolic blood pressure (DBP), medication, such as anti-diabetic agents and anti-obesity drugs, or alcohol consumption among the groups. The percentage of patients administered statins was significantly higher in the 35- to 64-year-old group than in the other age groups (Table 2, left column).

When patients were categorized by sex, BW, and waist circumference (WC) were significantly higher in males than in females, whereas percent body fat was significantly higher in females than in males. Serum aspartate transaminase (AST), ALT, γ-glutamyltransferase (γ-GTP), and triglyceride (TG) levels were significantly higher in males than in females. In contrast, the platelet count and serum levels of leptin, HDL-C, and adiponectin were significantly higher in females than in males. The percentage of females who never consumed alcohol was significantly higher than that of males. The percentage of males who had been administered statins was significantly higher than that of females. No significant differences were observed in the FIB4 index between males and females (Table 2, right column).

### Correlation between BW loss and changes in the FIB4 index

Three hundred and thirty-eight patients with obesity received weight reduction therapy for 12 months according to a previously reported protocol^17^. Among these patients, 41 (12%), 41, and 53 (16%) patients successfully reduced their BW by more than 5% from baseline BW after 3, 6, and 12 months (M) of weight reduction therapy, respectively. At each timepoint during weight reduction therapy, overlapping and non-overlapping cases were included (Table 3). Data of overlapping cases at more than two timepoints are shown in Supplementary Table 1.

**Table 3:** Body weight and the FIB4 index of patients who achieved their weight loss goals.

	3 M (n = 41)	6 M (n = 41)	12 M (n = 53)	p value
BW (kg)	72.4 (54.6–153.7)	79.3 ± 15.1	72.4 (53.3–149.0)	0.298
ΔBW loss (kg)	6.6 (3.6–22.4)	7.7 (5.0–38.2)	7.9 (3.5–42.1)	0.026*
FIB4 index	1.15 ± 0.56	0.97 (0.28–2.58)	1.08 ± 0.53	0.636
ΔFIB4 index	0.68 ± 0.29	0.10 ± 0.28	0.78 (− 0.83–0.67)	0.688

Patients successfully reduced their BW by more than 5% from baseline BW after 3, 6, and 12 months (M) of weight reduction therapy.

Data are expressed as the mean ± SD or medians (min, max).

Asterisks indicate a significant difference (p < 0.05) by the Kruskal–Wallis test.

Table 3 shows the BW and FIB4 index of patients who achieved their weight loss goals after 3, 6, and 12 M. ΔBW loss significantly increased in a time-dependent manner (p = 0.026). However, no significant difference was observed in ΔFIB4 index among the three time points examined.

We investigated the correlation between ΔBW loss and ΔFIB4 index after 3, 6, and 12 M of weight reduction therapy. We categorized patients into three groups by age, two groups by sex, and with or without alcohol consumption, and examined the correlation between ΔBW loss and ΔFIB4 index in each group (Table 4).

**Table 4:** Correlations between the amount of weight lost and changes in the FIB4 index.

	3 M (n = 41)		6 M (n = 41)		12 M (n = 53)	
	r	p	r	p	r	p
Total	−0.342	0.029*	−0.094	0.561	0.298	0.03*
Age ≤ 35 years	0.027	0.973	0.5	0.667	0.144	0.735
36 years ≤ Age ≤ 64 years	−0.298	0.139	0.049	0.806	0.387	0.02*
65 years ≤ Age	−0.492	0.124	−0.429	0.337	−0.361	0.339
FIB4 index < 1.3	−0.338	0.073	−0.057	0.76	0.229	0.167
1.3 ≤ FIB4 index ≤ 2.67	−0.804	0.002*	−0.309	0.385	−0.161	0.567
Male	−0.291	0.274	0.001	0.997	0.19	0.374
Female	−0.225	0.28	−0.151	0.492	0.336	0.074
Alcohol consumption Yes	−0.309	0.355	0.286	0.344	0.061	0.83
Alcohol consumption No	−0.263	0.264	−0.125	0.611	0.339	0.078

Patients successfully reduced their BW by more than 5% from baseline BW after 3, 6, and 12 months (M) of weight reduction therapy. Alcohol consumption was defined as current alcohol habits via a self-report and physician’s interview.

Yes: almost every day or sometimes. No: never.

Asterisks indicate a significant difference (p < 0.05).

**Figure 1:**
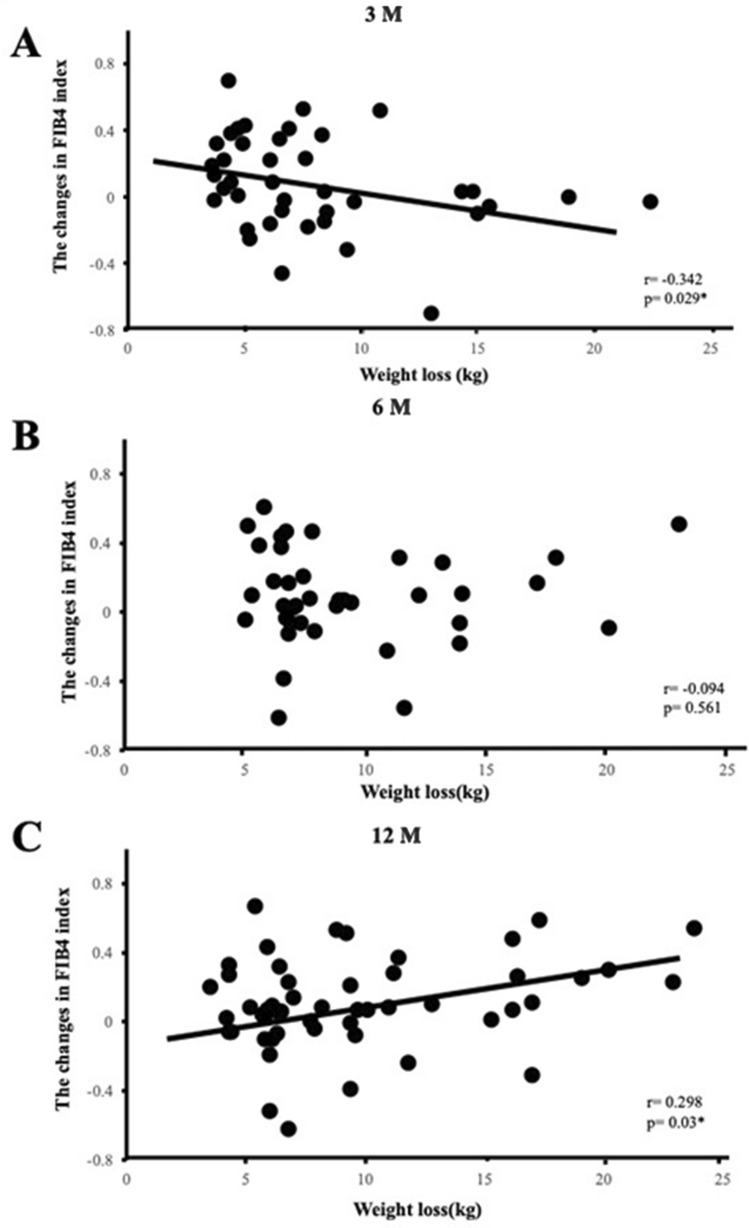
Correlation between the amount of weight lost and changes in the FIB4 index after 3 (**A**), 6 (**B**), and 12 (**C**) months (M) of weight reduction therapy. Patients reduced their body weight by more than 5% from the baseline at each time point. *p < 0.05.

**Figure 2:**
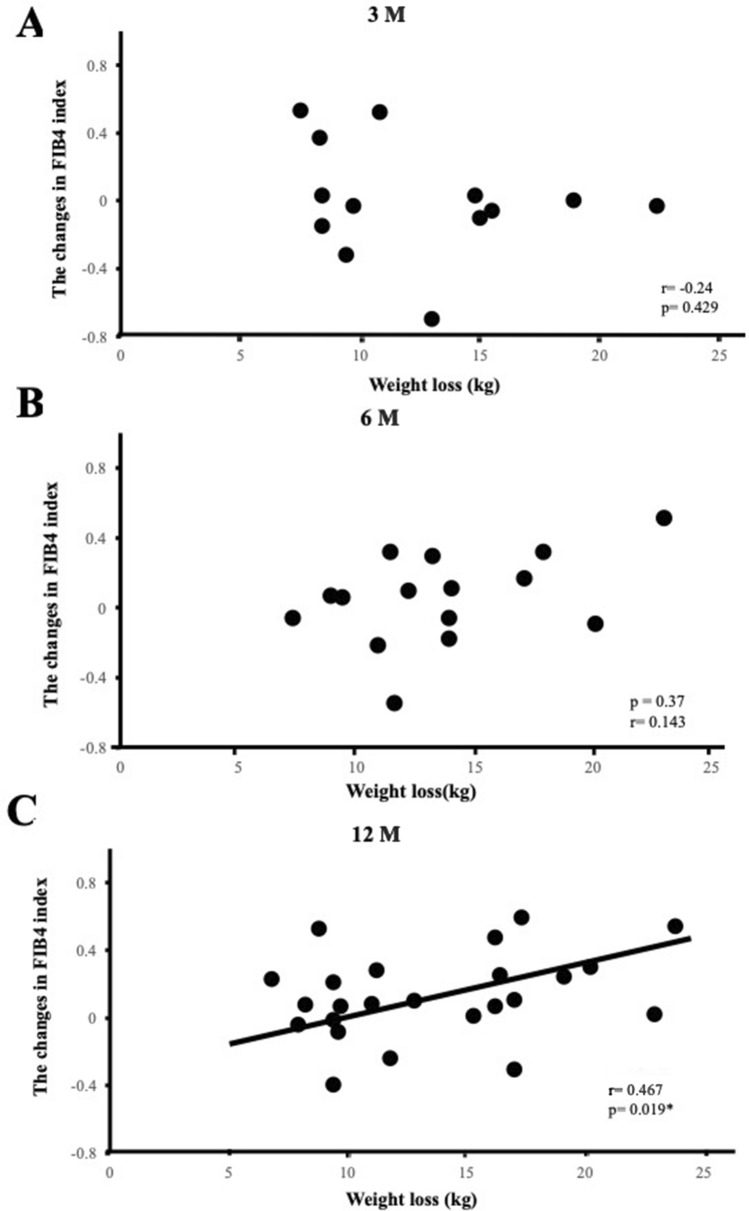
Correlation between the amount of weight lost and changes in the FIB4 index after 3 (**A**), 6 (**B**), and 12 (**C**) months (M) of weight reduction therapy. Patients reduced their body weight by more than 10% from the baseline at each time point. *p < 0.05.

A previous study reported that the liver fibrosis stage, which was histologically evaluated, improved by 1 stage in 45% of patients who received weight reduction therapy and achieved BW loss of more than 10% from the baseline, while it remained stable in the other 55%^9^. To examine the effects of BW loss in different liver fibrosis stages, we categorized patients into three groups by the baseline FIB4 index (Table 5). Previous studies reported that a FIB4 index ≥ 1.3 reflected liver fibrosis stages F2-F4, which indicated significant fibrosis, whereas a FIB4 index ≥ 2.67 reflected liver fibrosis stages F3-F4, namely, severe fibrosis^13,14^. Even though there were 9 patients with a FIB4 index > 2.67 (Table 5), none had successfully reduced their BW by more than 5% from the baseline. After 3 M of weight reduction therapy, a negative correlation (r = −0.342, p = 0.029) was observed between ΔBW loss and ΔFIB4 index in all 41 patients (Table 4, Fig. 1A). A negative correlation was also noted between ΔBW loss and ΔFIB4 index in the group with a FIB4 index of 1.3 to 2.67 (r = −0.804, p = 0.002) (Table 4). No correlations were found after 6 M (Table 4, Fig. 1B). However, after 12 M, a positive correlation was noted between ΔBW loss and ΔFIB4 index in all 53 patients (Table 4, Fig. 1C) and in the 36- to 64-year-old group (r = 0.298, p = 0.03, r = 0.387, p = 0.02, respectively) (Table 4). Furthermore, when we focused on patients who successfully reduced their BW by more than 10% from baseline BW after 3 and 6 M of weight reduction therapy, no correlation was observed between ΔBW loss and ΔFIB4 index (Fig. 2A,B), whereas a positive correlation was detected after 12 M (r = 0.467, p = 0.019) (Fig. 2C).

**Table 5:** Baseline characteristics of patients categorized by the FIB4 index.

Variable	FIB4 index < 1.3 (n = 258)	1.3 ≤ FIB4 index ≤ 2.67 (n = 71)	2.67 < FIB4 index (n = 9)	p value
Age (years)	46 ± 12.2	65 (38–82)	66 ± 6.6	< 0.001*
Male/Female	112/146	37/34	6/3	–
BW (kg)	81.5 (53–168)	74.0 (54.0–128.1)	74.7 ± 6.8	0.001*
BMI (kg/m^2^)	30.5 (25.0–56.1)	28.6 (25.0–49.9)	28.4 ± 2.4	0.009*
WC (cm)	100 (71–150)	98 (81–150)	97.8 ± 7.8	–
% fat (%)	36.8 (19.6–64.5)	31.1 (16.8–64.2)	29.4 ± 7.8	< 0.001*
SBP (mmHg)	141 ± 19.1	140 (96–191)	138 ± 12.6	–
DBP (mmHg)	86 ± 12.1	83 ± 10.9	81 ± 6.5	–
FPG (mg/dl)	98 (66–407)	112 (68–354)	124 ± 47.6	0.017*
HbA1c (%)	5.6 (4.5–11.6)	6.0 (4.9–9.3)	6.3 ± 1.1	0.035*
IRI (μU/ml)	14.9 (3.9–398.8)	14.8 (2.0–180.3)	15.6 ± 9.4	–
HOMA-R	3.82 (0.88–170.3)	4.10 (0.55–62.8)	4.83 ± 3.54	–
HOMA-β	149 (15–3048)	124 (15–832)	87 (30–1001)	–
AST (IU/l)	23 (8–130)	28 (13–71)	66.5 (33–667)	< 0.001*
ALT (IU/l)	32 (6–171)	28 (11–122)	52 (27–315)	–
Plt (10^9^/L)	272 (149–3010)	195 (130–340)	137 ± 38	< 0.001*
γ-GTP (IU/l)	40 (9–269)	39 (11–376)	110 (90–2527)	0.003*
TG (mg/dl)	150 (47–1766)	148 (58–951)	185 ± 146	–
HDL-C (mg/dl)	54 (21–103)	55 (18–103)	64 ± 6.5	–
LDL-C (mg/dl)	129 ± 30	127 (73–224)	110 ± 33.1	–
Leptin (ng/ml)	13.6 (0.96–135.7)	13.1 (1.40–88.4)	10.1 ± 5.2	–
Adiponectin (μg/ml)	5.93 (2.06–36.1)	7.11 (1.29–17.0)	6.92 ± 2.7	0.012*
FIB4 index	0.76 (0.73–1.30)	1.75 (1.32–2.56)	3.15 (2.75–18.5)	< 0.001*
**Concomitant medication at baseline, n (%)**				
Antidiabetic drugs, n (%)	59 (23)	16 (23)	2 (22)	–
Antilipidemic drugs, n (%)	116 (45)	35 (49)	3 (33)	–
Antiobesity drugs, n (%)	17 (7)	2 (3)	1 (11)	–
Alcohol consumption				0.019*
Never, n (%)	118 (58)	27 (50)	2 (29)	
Sometimes, n (%)	55 (27)	10 (19)	2 (29)	
Every day, n (%)	29 (15)	17 (31)	3 (42)	

Data are expressed as the mean ± SD, medians (min, max), or percentages.

Asterisks indicate a significant difference (p < 0.05).

### Effects of weight reduction therapy on components of the FIB4 index calculation formula and metabolic parameters

We examined the effects of weight reduction therapy on changes in the components of the FIB4 index calculation formula and metabolic parameters (Table 6). No correlations were observed between changes in three components (serum AST and ALT levels and the platelet count) of the FIB4 index formula and the amount of weight lost at the indicated time points (Table 6). There was also no correlation between baseline age and the amount of weight lost (Table 6).

**Table 6:** Correlations between the amount of weight lost and changes in the platelet count and metabolic parameters.

	3 months (M) (n = 41)		6 M (n = 41)		12 M (n = 53)	
	r	p	r	p	r	p
Age	0.085	0.599	0.016	0.923	−0.149	0.289
ΔAST	−0.175	0.274	0.113	0.481	0.175	0.21
ΔALT	−0.177	0.268	0.081	0.614	0.101	0.473
ΔPlt	0.213	0.182	0.19	0.234	−0.249	0.072
ΔAST/√ALT	−0.118	0.461	0.26	0.101	0.282	0.041*
Δ1/Plt	−0.335	0.032*	−0.205	0.198	0.175	0.209
ΔAPRI	−0.297	0.059	0.019	0.905	0.212	0.127
ΔWC	−0.301	0.079	−0.385	0.019*	−0.627	< 0.001*
Δ%fat	−0.369	0.041*	−0.597	< 0.001*	−0.597	< 0.001*
ΔFPG	0.05	0.757	0.157	0.327	−0.087	0.537
ΔIRI	−0.109	0.588	−0.148	0.454	0.187	0.276
ΔHOMA-R	−0.095	0.638	−0.116	0.558	0.161	0.348
ΔHOMA-β	−0.059	0.77	−0.115	0.561	0.166	0.341
ΔHbA1c	−0.199	0.226	−0.187	0.255	−0.31	0.027*
ΔTG	−0.215	0.182	−0.08	0.629	−0.087	0.541
ΔHDL-C	0.104	0.527	−0.054	0.746	−0.133	0.348
ΔLDL-C	−0.11	0.51	−0.211	0.198	0.053	0.708
ΔAdiponectin	0.189	0.263	0.312	0.547	0.299	0.155
ΔLeptin	−0.353	0.035*	−0.371	0.468	−0.573	0.003*

Asterisks indicate a significant difference (p < 0.05).

We examined AST/√ALT and the inverse number of the platelet count (1/Plt), which are components of the FIB4 index formula. After 3 M, a negative correlation was found between ΔBW loss and changes in 1/Plt (Δ1/Plt). After 12 M, a positive correlation was noted between ΔBW loss and changes in AST/√ALT (ΔAST/√ALT). We also analyzed the AST to platelet ratio index (APRI), another non-invasive scoring system for the assessment of hepatic fibrosis^24^. No correlations were observed between ΔBW loss and changes in APRI (ΔAPRI) at any of the indicated time points after weight loss (Table 6).

After 3 and 12 M of weight reduction therapy, a negative correlation was noted between changes in serum leptin levels (ΔLeptin) and ΔBW loss (Table 6). A correlation was observed between ΔBW loss and changes in WC (ΔWC) after 6 and 12 M, but not after 3 M (Table 6). Moreover, after 12 M, a correlation was detected between changes in hemoglobin A1c (HbA1c) levels (ΔHbA1c) and ΔBW loss (Table 6).

## Discussion

The present study investigated time-dependent changes in the FIB4 index in patients with obesity receiving weight reduction therapy. We hypothesized that ΔBW loss negatively correlates with ΔFIB4 index. However, the results obtained demonstrated that our hypothesis was incorrect (Table 4). No correlation was observed between ΔBW loss and ΔFIB4 index in patients whose data were examined at any two time points; however, correlation coefficients increased in a time-dependent manner. Furthermore, there were only 10 patients whose data were examined at all three time points and, thus, there was insufficient power for a statistical analysis (Supplementary Table 1).

To understand these antithetical results, we investigated the effects of BW loss on changes in metabolic parameters (Table 6). A decrease in serum leptin levels correlated with weight loss, indicating a reduction in body fat^25,26^. We also found that ΔWC and ΔHbA1c correlated with ΔBW loss. Collectively, these results suggest that weight reduction therapy improved the metabolic status.

Although the components of the FIB4 index formula could be affected by BW loss in previous studies^27–30^, no correlation was observed between changes in the three components of the FIB4 index formula (AST, ALT and platelet count) and ΔBW loss at any of the indicated timepoints in the current study (Table 6). Nonetheless, changes in AST/√ALT, which is a part of the FIB4 index formula, was positively correlated with the amount of weight loss.

Age, one of the components of the formula, may also have affected the present results. Patients who achieved their weight loss goals after 3 and 6 M remained at the same age during the period; however, after 12 M, 38 out of the 53 patients (72%) were one year older, which may have increased the FIB4 index (Tables 4, 6) ^21^. Age has been shown to significantly affect the FIB4 index^19^. Serum ALT levels decrease with age even after adjustments for sex and the parameters used to diagnose metabolic syndrome^31,32^. The platelet count also decreases with age^33^. Since age is a numerator and both ALT and the platelet count compose the denominator of the FIB4 index formula, we speculated that this also may have affected an increased FIB4 index at 12 M. Furthermore, we found a higher baseline FIB4 index in patients older than 65 years, suggesting an overestimation of the FIB4 index, which is consistent with previous findings^19,20^. Moreover, we also found that the positive correlation between ΔBW loss and ΔFIB4 index after 12 M of weight reduction therapy focused on patients who successfully reduced their BW by more than 10% from baseline BW (Fig. 2C). Since it was reported that liver fibrosis was significantly and histologically improved in these patients^9^, it is unlikely that the positive correlation may reflect exacerbated liver fibrosis. Thus, collectively, we concluded that the positive correlation between ΔBW loss and ΔFIB4 index after 12 M of weight reduction therapy may not mean that BW loss exacerbates liver fibrosis.

There are a number of limitations that need to be addressed. Since detailed information was not obtained from patients on alcohol consumption, the present study may have included patients with alcohol-related fatty liver, which often coexists with NAFLD^34^. However, alcohol consumption by patients in the present study was unlikely to have affected the relationship between the FIB4 index and liver transaminases because the baseline FIB4 index correlated with serum AST levels, but not ALT levels regardless of alcohol consumption (Supplementary Table 2). Liver steatosis and fibrosis were not assessed by imaging modalities. Liver steatosis may be present in more than 80% of patients with BMI > 28^35^, and a positive correlation has been reported between BMI and fatty liver^36^. Furthermore, liver biopsy was not performed. Nevertheless, since we examined patients who achieved BW loss of more than 5% from the baseline, we speculated that histological improvements occurred in patients with liver steatosis, as previously reported^8,9^.

We examined time-dependent changes in the FIB4 index in patients with obesity receiving weight reduction therapy for 12 months. The FIB4 index is a practically easy and useful tool for detecting and evaluating liver fibrosis. However, the present study revealed changes in the FIB4 index itself and the correlation between ΔBW loss and ΔFIB4 index during weight reduction therapy, which were mainly due to time-dependent changes in the four components (serum AST and ALT levels, the platelet count, and age) of the FIB4 index formula. Thus, we concluded that the FIB4 index is useful and reliable to assess liver fibrosis until 3 M during weight reduction therapy. However, after 3 M, we should recognize that the FIB4 index may not reflect liver status. Therefore, it is important to consider this characteristic of the FIB4 index as a limitation when assessing liver fibrosis in patients with obesity receiving weight reduction therapy.

## Materials and methods

### Subjects

A total of 508 Japanese outpatients with obesity (231 males and 277 females, mean age 49 years) were consecutively enrolled in JOMS between 2005 and 2010^17^. JOMS was registered in the University Hospital Medical Information Network Clinical Trial Registry (UMIN-CTR) system (ID: UMIN000000559). Approval for the study was obtained from the ethics committee for human research at Kyoto Medical Center (approval number: 14–034). The study was carried out in accordance with the principles of the Declaration of Helsinki and the Ethical Guidelines for Medical and Health Research Involving Human Subjects. Written informed consent was obtained from all participants. The study was designed to assess the characteristics of metabolic syndrome and the success rate and effects of weight reduction therapy with diet and exercise guidelines for the prevention of cardiovascular disease (CVD) in Japanese patients with obesity. We recruited subjects with obesity with BMI > 25 kg/m^2^. Subjects were excluded if they had a previous history of severe liver dysfunction, CVD, other vascular diseases, or apparent renal disease^8,17^.

### Data collection and laboratory assay

Height and weight were measured, followed by the calculation of BMI. WC was measured at the level of the umbilicus in the standing position. The total body fat percentage was assessed by the bio-impedance method using the Tanita body fat analyzer (Tanita Corporation, Tokyo, Japan). SBP and DBP were measured twice with an automatic electronic sphygmomanometer (BP-103iII; Nippon Colin, Komaki, Japan).

Blood was collected in the morning after a 12-h fast without the intake of medication to measure the platelet count and the fasting levels of plasma glucose, HbA1c, IRI, serum LDL-C, HDL-C, TG, AST, ALT, γ-GTP, leptin, and adiponectin. HOMA-R and HOMA-β were evaluated by the homeostasis model assessment.

IRI was measured using an enzyme immunoassay (Tosoh, Tokyo, Japan). Serum adiponectin levels were assessed with enzyme-linked immunosorbent assays (Assay Pro, St. Charles, USA, and Otsuka Pharmaceutical, Tokyo, Japan, respectively). Serum leptin levels were evaluated using a radioimmunoassay (Linco Research, St. Charles, USA).

The FIB4 index was calculated using the following equation: Age (years) × AST (IU/L)/(√ALT (IU/L) × Platelet count (10^9^/L))^13^. Current alcohol consumption was defined as current alcohol habits via a self-report and physician’s interview. Patients selected from three answers: almost every day, sometimes, and never^37^.

### Weight reduction therapy

Three hundred and thirty-eight Japanese outpatients with obesity (155 males and 183 females, mean age 50.0 years, mean BMI 31.3) enrolled in JOMS and received weight reduction therapy through lifestyle modifications to reduce energy intake and increase physical activity for 12 M. The prescribed diet consisted of 25 kcal/kg of the ideal body weight per day, and subjects were instructed to exercise for at least 30 min at a moderate intensity at least 3 days/week^38,39^. We explained to the patients that walking for 30 min at a pace of about 100 steps per minute is an exercise at a moderate intensity. Before and 3, 6, and 12 M after weight reduction therapy, we measured BMI, WC, percent fat, SBP/DBP, and laboratory tests and calculated the FIB4 index for each patient. The administration of anti-diabetic and anti-hyperlipidemic agents remained unchanged during the observation periods. The diet and exercise records of subjects were monitored to confirm compliance. Since hepatic histological improvements were previously reported in NAFLD patients who achieved BW loss of more than 5% from the baseline^8^, we defined patients with BW loss of 5% or more from the baseline as successful.

### Statistical analysis

Continuous and parametric data are presented as the mean ± standard deviation, and non-parametric data as medians (min–max). Categorical variables were summarized as a percentage.

The Chi-squared test was used to examine the distribution of categorical values between groups. The Student’s two-tailed *t*-test was employed to evaluate differences in DBP between males and females. The Mann–Whitney test was performed for other comparisons between the two groups. The Kruskal–Wallis test was used to compare mean values among three categorized groups.

Pearson’s correlation test was conducted to examine the relationships between ΔBW loss and ΔFIB4 index after 3 and 12 M of weight reduction therapy, and ΔBW loss and ΔPlt after 3 M in the ≤ 35-year-old group. Spearman’s correlation test was used to investigate other relationships.

The Shapiro–Wilk test was performed to establish whether continuous variables were normally distributed. *p* < 0.05 was considered to be significant. All statistical analyses were performed using SPSS 28.0 for Macintosh (SPSS Inc., Chicago, IL, USA).

## Supplementary Information

Supplementary Tables.
