# Supplementary material for: Analysis of time-dependent changes in the FIB4 index in patients with obesity receiving weight reduction therapy
Source: Sci Rep. 2022 Sep 8;12:15219. doi: 10.1038/s41598-022-19420-0 (PMC9458712; doi:10.1038/s41598-022-19420-0)
Supplement: Supplementary file 1 — Supplementary Tables. [file 41598_2022_19420_MOESM1_ESM.pdf]

Supplementary information

Analysis of time-dependent changes in the FIB4 index in patients with obesity receiving weight reduction therapy

Shiori Kawai, Hajime Yamakage, Kazuhiko Kotani, Mitsuhiko Noda,
Noriko Satoh-Asahara, and Koshi Hashimoto

Address all correspondence to:
Koshi Hashimoto, M.D., Ph.D.
Department of Diabetes, Endocrinology and Hematology,
Dokkyo Medical University Saitama Medical Center,
2-1-50 Minami-Koshigaya, Koshigaya, Saitama 343-8555, Japan
Tel/Fax: +81-48-965-1111/8253. E-mail: k-hashi@dokkyomed.ac.jp

Supplementary Table 1.

Correlations between the amount of weight lost and changes in the FIB4 index in overlapping cases at indicated time points.

	3 and 6 months (M) (n=18)		3 and 12 M (n=17)		6 and 12 M (n=21)		every time point (n=10)	
	r	p	r	p	r	p	r	p
3 M	-0.445	0.064	-0.21	0.419	-	-	-0.43	0.214
6 M	-0.117	0.645	-	-	0.392	0.079	0.091	0.803
12 M	-	-	-0.01	0.97	0.416	0.06	-0.043	0.907

Supplementary Table 2. Correlations between the baseline FIB4 index and components of the FIB4 index calculation formula categorized by age, sex, and alcohol consumption

	Age ≤35 years (n=54)		36 years ≤Age ≤ 64 years (n=221)		65 years ≤Age (n=63)	
	r	p	r	p	r	p
Age	0.371	0.006*	0.688	<0.001*	0.235	0.064
AST	0.637	<0.001*	0.447	<0.001*	0.558	<0.001*
ALT	0.497	<0.001*	0.174	0.009*	0.247	0.051
Plt	-0.589	<0.001*	-0.718	<0.001*	-0.735	<0.001*

	Male (n=155)		Female (n=183)	
	r	p	r	p
Age	0.866	<0.001*	0.824	<0.001*
AST	0.19	0.018*	0.382	<0.001*
ALT	-0.147	0.068	0.019	0.797
Plt	-0.707	<0.001*	-0.749	<0.001*

	Alcohol consumption Yes (n=116)		Alcohol consumption No (n=147)	
	r	p	r	p
Age	0.859	<0.001*	0.831	<0.001*
AST	0.37	<0.001*	0.24	0.003*
ALT	0.038	0.683	-0.055	0.507
Plt	-0.701	<0.001*	-0.711	<0.001*

Alcohol consumption was defined as current alcohol habits via a self-report and physician’s interview. Yes: almost every day or sometimes. No: never.

Asterisks indicate a significant difference (p <0.05).
